# Cholecalciferol Supplementation Induced Up-Regulation of *SARAF* Gene and Down-Regulated miR-155-5p Expression in Slovenian Patients with Multiple Sclerosis

**DOI:** 10.3390/genes14061237

**Published:** 2023-06-08

**Authors:** Saša Gselman, Tanja Hojs Fabjan, Anja Bizjak, Uroš Potočnik, Mario Gorenjak

**Affiliations:** 1 Clinic of Neurology, University Clinical Centre Maribor, 2000 Maribor, Slovenia; sasa.gselman@gmail.com (S.G.); tanja.hojsfabjan@ukc-mb.si (T.H.F.); 2 Department of Neurology, Faculty of Medicine, University of Maribor, 2000 Maribor, Slovenia; 3 Center for Human Molecular Genetics and Pharmacogenomics, Faculty of Medicine, University of Maribor, 2000 Maribor, Slovenia; anja.bizjak2@um.si; 4 Laboratory of Biochemistry, Molecular Biology and Genomics, Faculty of Chemistry and Chemical Engineering, University of Maribor, 2000 Maribor, Slovenia; 5 Department for Science and Research, University Clinical Centre Maribor, 2000 Maribor, Slovenia

**Keywords:** multiple sclerosis, relapsing–remitting multiple sclerosis, micro RNA, miR-155-5p, *SARAF* gene

## Abstract

Multiple sclerosis is a common immune-mediated inflammatory and demyelinating disease. Lower cholecalciferol levels are an established environmental risk factor in multiple sclerosis. Although cholecalciferol supplementation in multiple sclerosis is widely accepted, optimal serum levels are still debated. Moreover, how cholecalciferol affects pathogenic disease mechanisms is still unclear. In the present study, we enrolled 65 relapsing–remitting multiple sclerosis patients who were double-blindly divided into two groups with low and high cholecalciferol supplementation, respectively. In addition to clinical and environmental parameters, we obtained peripheral blood mononuclear cells to analyze DNA, RNA, and miRNA molecules. Importantly, we investigated miRNA-155-5p, a previously published pro-inflammatory miRNA in multiple sclerosis known to be correlated to cholecalciferol levels. Our results show a decrease in miR-155-5p expression after cholecalciferol supplementation in both dosage groups, consistent with previous observations. Subsequent genotyping, gene expression, and eQTL analyses reveal correlations between miR-155-5p and the *SARAF* gene, which plays a role in the regulation of calcium release-activated channels. As such, the present study is the first to explore and suggest that the *SARAF* miR-155-5p axis hypothesis might be another mechanism by which cholecalciferol supplementation might decrease miR-155 expression. This association highlights the importance of cholecalciferol supplementation in multiple sclerosis and encourages further investigation and functional cell studies.

## 1. Introduction

Multiple sclerosis is a common immune-mediated inflammatory and demyelinating disease of the central nervous system, which makes it the leading cause of disability among young adults [1,2]. Diagnosis in patients with suggestive clinical presentation is made using the McDonald’s criteria of dissemination in space and time [3]. Nevertheless, sometimes there is a necessity for increasing the diagnostic confidence of imaging with oligoclonal bands or free light chains in cerebrospinal fluid [4]. Multiple sclerosis is a complex disease that results from genetic, epigenetic, and environmental factors, which trigger autoimmune mechanisms, which, in turn, cause demyelination, subsequent axonal damage, and neurodegeneration [5]. Cholecalciferol, generally known also as vitamin D, is one of the main environmental factors involved in the pathogenesis of multiple sclerosis and it was previously shown that lower cholecalciferol serum levels are associated with an increased risk of developing multiple sclerosis and greater disease activity [6,7]. However, the exact impact of cholecalciferol supplementation on disease activity remains unclear and optimal serum levels and supplementation doses remain controversial.

In the last decade, research data are showcasing that cholecalciferol serum levels are associated with genes or genetic loci involved in cholecalciferol metabolism, transport, and elimination [8]. These findings are leading to further research of these genetic loci because of well-known correlations between the disease prevalence and geographic latitude, which can be explained by the effect of ultraviolet radiation or cholecalciferol serum levels [7,9,10,11]. However, the open question remains if genetic loci associated with cholecalciferol serum levels are also related to disease susceptibility [12].

Additionally, epigenetic factors such as small non-coding microRNA molecules (miRNAs) are dysregulated in many autoimmune diseases including multiple sclerosis [13,14,15,16,17,18]. They are involved in the post-transcriptional regulation of gene expression [13]. MiR-155 is one of the main pro-inflammatory miRNAs playing a role in the pathogenesis of multiple sclerosis [16]. MiR-155 is encoded by the host gene *MIR155HG* [16]. Overexpression of miR-155 has been noticed in active brain lesions and also in peripheral blood cells [16,19]. The exerted pro-inflammatory effect of miR-155 has an impact on the infiltration of peripheral immune cells, causes demyelination via microglia activation, phagocytosis of myelin by macrophages, differentiation of T-cells, and also contributes to increased permeability of the blood–brain barrier [19,20,21]. 

The roles of miRNA molecules in multiple sclerosis are flagged and proposed as promising diagnostic biomarkers [13,22]. Furthermore, it was shown that miRNAs can accurately differentiate patients with relapsing–remitting multiple sclerosis (RRMS) from healthy controls [23]. Additionally, it was also shown that the biological active form of vitamin D (1,25(OH)_2_D_3_) modulates and suppresses inflammation by down-regulating miR-155 expression [24], which, in turn, establishes a bridge between miR-155 and cholecalciferol. Nevertheless, studied and established associations between co-regulation of genetic, epigenetic (miRNAs), and cholecalciferol supplementation are scarce.

Therefore, in order to investigate the possible interplay between cholecalciferol, genetic, and epigenetic factors, we conducted a double-blind randomized study during winter time on a specific cohort of Slovenian relapsing–remitting multiple sclerosis (RRMS) patients with the aim to explore the continuum of connections between miR-155-5p expression, cholecalciferol supplementation, genetic variants, and miR-155-5p genetic targets as follows:

1. Measure the miR-155-5p expression in peripheral blood mononuclear cells (PBMCs);
2. Profile the genome for variants associated with cholecalciferol uptake;
3. Stringently select genetic targets of miR-155-5p and extract genetic variants associated with cholecalciferol pathways;
4. Calculate and assess expression quantitative trait loci (eQTLs) between aforementioned genetic variants, miR-155-5p, and miR-155-5p target genes;
5. Identify target genes where both eQTLs are observed and measure the expression of the corresponding gene.

## 2. Materials and Methods

### 2.1. Subjects

We enrolled 65 patients with diagnosed relapsing–remitting multiple sclerosis from the Department of Neurologic Diseases University Clinical Centre Maribor, Slovenia. The patients were double-blindly divided into two groups for a low dose (1000 IU per day) and a high dose (4000 IU per day) of cholecalciferol supplementation. All patients were aged 18–60 years, were on immunomodulatory therapy, and had an Expanded Disability Status Scale (EDSS) value of <5. The demographic data before supplementation are summarized in Table 1. Exclusion criteria were taking cholecalciferol supplementation during three month wash-out period before the study, pregnancy or breastfeeding, relapse of the disease or corticosteroid treatment in the last month, any viral or inflammatory disease at the enrollment, renal dysfunction, high calcium or parathyroid hormone, change in the immunomodulatory therapy in the last three months before enrollment, concomitant autoimmune diseases and anamnesis of hyperparathyroidism, liver disease, tuberculosis, sarcoidosis, or kidney stones. The patients were receiving oil suspension of cholecalciferol (Fresuvit D_3_, Fresenius Kabi Austria GmbH) during four winter months from November until February. Before and after cholecalciferol supplementation, EDSS and the Multiple Sclerosis Functional Composite (MSFC) score were assessed. Sun exposure questionnaire [25] and short questionnaire for assessment of dietary vitamin D intake [26] diaries were filled out during the study. 

### 2.2. Sample Collection

At the time of enrollment and at the end of the four-month period, we collected 12 mL peripheral venous blood and placed it into potassium salt of ethylene diamine tetra acetic acid (K_2_EDTA) tubes for mononuclear cell isolation and placed 3 × 6 mL into serum tubes for blood biochemistry. Measured biochemistry parameters were serum cholecalciferol level using Cobas e601 apparatus (Roche Diagnostics, Penzberg, Germany), creatinine, calcium, phosphate, C-reactive protein using Siemens Dimension Vista apparatus (Siemens HealthCare Diagnostics, Newark, DE, USA), and parathyroid hormone using Cobas e411 apparatus (Roche).

### 2.3. DNA, mRNA, and miRNA Extraction

DNA and mRNA were extracted from one portion of PBMCs using TRI-reagent (Merck, Darmstadt, Germany) according to the manufacturer’s instructions. MiRNA was extracted and purified from the other portion of PBMCs using miRNeasy Mini Kit (Qiagen, Germantown, MD, USA) according to the manufacturer’s instructions. Purity and concentration of nucleic acids were assessed using Synergy 2 spectrophotometer (Biotek, Winooski, VT, USA) and Qubit (Thermo Fisher, Waltham, MA, USA).

### 2.4. MiR-155-5p RT-qPCR

MiRNA RT-qPCR was performed using miRCURY LNA RT Kit, miRCURY LNA SYBR Green PCR Kit, and miRCURY LNA miRNA PCR Assay (Qiagen) according to the manufacturer’s instructions. PCR assays used were miR-155-5p as the target miRNA and SNORD49A and SNORD38B as reference miRNAs. Target miRNA was normalized to the geometric mean of reference miRNAs and expressed in linear form of 2^−ΔCt^ [27]. Statistical analysis of miR-155-5p before and after cholecalciferol supplementation and between groups was carried out using Wilcoxon paired samples rank test and Mann–Whitney U test. Additionally, linear mixed models using lme4 R package [28] were performed and fitted using the blocking technique, where a blocking factor was set as a random variable and time point, cholecalciferol dosage, age, sex, and cholecalciferol levels were used as covariates to correct for.

### 2.5. Genotyping, Imputation, and Association Analysis

DNA was genotyped using genotyping microarray Infinium Global Screening Array (GSA_24v3) and iScan apparatus (Illumina, San Diego, CA, USA) according to manufacturer’s instructions. Quality control of raw genotype data was performed as previously described [29]. Genotype imputation was carried out using the Michigan imputation server Minimac3 genotype imputation algorithm and using the Haplotype Reference Consortium (HRC r1.1 2016) reference panel and SHAPEIT v2.r790 phasing [30]. Association analysis was performed with change (Δ) in cholecalciferol values (post minus pre) as an outcome variable using linear regression implemented in PLINK 2.0 (www.cog-genomics.org/plink/2.0/, accessed on 17 May 2021) [31]. To account for the non-normally distributed outcome variable, a two-step inverse normal transformation was performed using FRGEpistasis R package [32]. Regression was corrected for sex, age, sun exposure, cholecalciferol dosage, and first four principal components, and was performed using imputed allelic dosages. 

### 2.6. Integration of Genomics to miRNA-155-5p Targets

Variants identified in association analysis were integrated with miRNA-155-5p target genes, which were listed in miRWalk database (http://mirwalk.umm.uni-heidelberg.de/, accessed on 9 December 2022). Only target genes with a miRNA-gene-binding probability of 1.00 and with additional experimental MiRTarBase validation were selected [33,34]. Binding site nucleotide sequence seeds for miRNA on selected target genes were identified on GRCh37 DNA nucleotide sequences using BLAST (https://blast.ncbi.nlm.nih.gov/Blast.cgi, accessed on 9 December 2022) [35]. Genomic variants ±200 bp (10 times the average of the binding region length in order to expand the search region) from miRNA binding seeds were extracted from association analysis and further analyzed. Evidence of statistically significant signal was considered for variants with adjusted *p*-value < 0.05. Genotypes of statistically significant variants were extracted and assessed for eQTL with miR-155-5p expression using linear mixed models using lme4 R package [28] and fitted using blocking technique where blocking factor was set as a random variable and time point, sex, age, dosage, and genotype were used as covariates to correct for. For variants with significant eQTLs with miR-155-5p, additional dominant and recessive models were tested. A statistical significant signal was considered at *p*-value < 0.05.

### 2.7. RT-qPCR Target Gene Validation

MiR-155-5p targets that were identified in miRNA genomic integration were validated using the reverse-transcription quantitative polymerase chain reaction (RT-qPCR) method. A total of 1 µg of extracted mRNA was transcribed into cDNA using a high-capacity cDNA reverse transcription kit (Thermo Fisher, Waltham, MA, USA). Nucleotide sequences for mRNA of target gene *SARAF* (NM_016127.6) were retrieved from the NCBI nucleotide database (https://www.ncbi.nlm.nih.gov/nuccore/, accessed on 19 December 2022) and primers were designed using IDT OligoAnalyzer tool (eu.idtdna.com/calc/analyzer, accessed on 19 December 2022). Nucleotide sequences for the *SARAF* gene were as follows: FW 5′—GTTTTGGCAGTGCTTTTACA—3′ and RV 5′—ACGAGTCTGAGAAGGGTGTT—3′. Primers were synthesized by Sigma (Merck, Darmstadt, Germany). Primers for the reference genes *ACTB* and *B2M* were obtained from a previous study [36]. RT-qPCR assays were carried out using LightCycler 480 SYBR Green I Master Mix and LightCycler 480 real-time thermocycler (Roche, Basel, Switzerland) according to manufacturer’s instructions. A total of 2 μL of 20-fold diluted cDNA (2.5 ng/μL) was used as a template for a single PCR reaction. Melting curves of each sample were analyzed after each run in order to confirm amplification specificity. Raw Ct values were obtained from independent technical duplicates for each sample and normalization of raw Ct values was carried out using the geometric mean of both reference genes and linear expression was calculated using 2^−ΔCt^ calculation [27] in order to allow statistical analyses. Statistical analysis of *SARAF* expression before and after cholecalciferol supplementation and between groups was carried out using Wilcoxon paired samples rank test and Mann–Whitney U test. Additionally, linear mixed models using the lme4 R package [28] were performed and fitted using the aforementioned blocking technique.

### 2.8. Statistical Analyses

Data were analyzed using R 4.1.3 environment (R Core Team 2020, Vienna, Austria). Statistical differences between nominal categorical variables were estimated using Fisher’s exact test. All continuous variables were first assessed for normality of distribution using the Kolmogorov–Smirnov test of normality. Statistical differences of continuous variables between two groups were assessed using Mann–Whitney U tests. Differences between two timepoints were assessed using Wilcoxon signed rank test. Correlations were estimated using Spearman rank correlations. The dosage effect of cholecalciferol supplementation was estimated using generalized linear models with Δ cholecalciferol values (post minus pre) as an outcome variable corrected for sex, age, and consumption of fish, milk, yogurt, margarine, and sun exposure.

## 3. Results

### 3.1. Estimation of Cholecalciferol Supplementation

**Figure 1:**
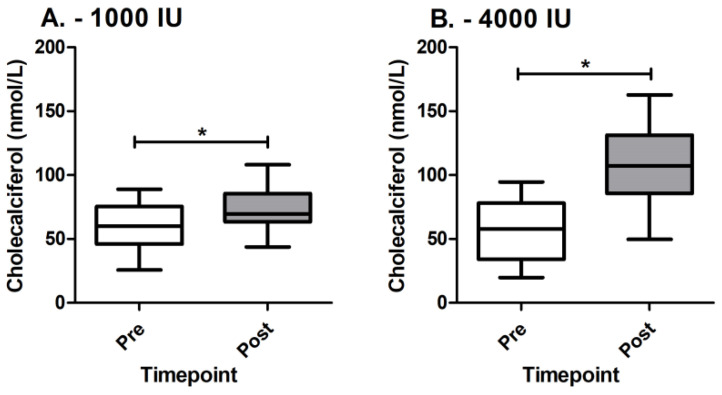
Cholecalciferol levels between groups before and after supplementation. (**A**) Cholecalciferol levels pre and post in 1000 IU group; (**B**) cholecalciferol levels pre and post in 4000 IU group; * denotes statistically significant difference.

First, we assessed the effect of cholecalciferol supplementation on blood serum levels. In both groups receiving 1000 IU and 4000 IU of cholecalciferol supplementation, we observe a statistically significant increase in cholecalciferol in serum (Figure 1A,B). In the group receiving 1000 IU, the rise in serum levels is from 59.3 ± 18 to 72.5 ± 16.2 nmol/L (*p* = 3.6 × 10^−5^) and in the group receiving 4000 IU, the rise in serum levels is from 56.2 ± 22 to 106.5 ± 32 nmol/L (*p* = 1 × 10^−6^). A between-groups difference is also observed after supplementation (*p* = 1.2 × 10^−5^). Additionally, fitted generalized linear models also show a statistically significant effect of the dosage on blood serum cholecalciferol levels (β: 39.98; *p* = 5.9 × 10^−4^).

### 3.2. MiR-155-5p Expression

**Figure 2:**
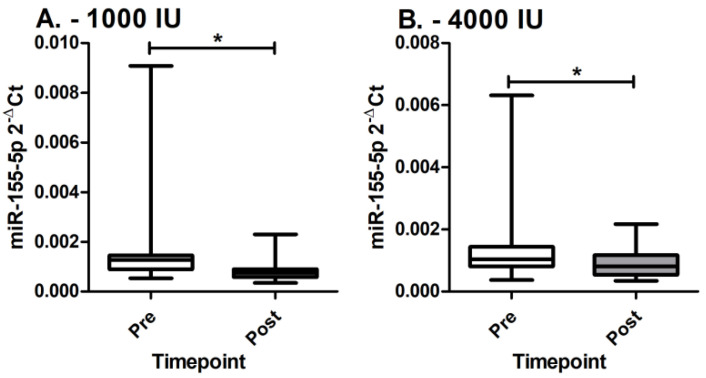
MiR-155-5p expression levels between groups before and after supplementation. (**A**) MiR-155-5p expression levels pre and post in 1000 IU group; (**B**) MiR-155-5p expression levels pre and post in 4000 IU group; * denotes statistically significant difference.

We observe that miR-155-5p expression in PBMCs statistically significantly decreases in both groups receiving 1000 IU and 4000 IU of cholecalciferol supplementation (Figure 2). In the group receiving 1000 IU of cholecalciferol supplementation, the miR-155-5p expression decreases from 0.0015 ± 0.0015 to 0.0008 ± 0.0004 (*p* = 1.53 × 10^−4^). In the group receiving 4000 IU of cholecalciferol supplementation, the miR-155-5p expression decreases from 0.0013 ± 0.0011 to 0.0009 ± 0.0004 (*p* = 0.021). Additionally, linear mixed models were applied in order to confirm the decrease between timepoints with correction for cholecalciferol dosage, age, sex, and cholecalciferol levels. We again observe that miR-155-5p levels statistically significantly decrease (F: 9.541; *p* = 0.0027). However, a between-groups difference is not observed after supplementation (*p* = 0.372).

### 3.3. MiR-155-5p Targets, Integration to Genomics, and eQTL Estimation

A total of 70 miR-155-5p targets were chosen from the miRWalk database and GRCh37 DNA nucleotide sequence seed correspondence was obtained (Table S1). Genomic variants in genomic regions ranging ±200 bp from validated miRNA binding seeds were extracted from association analysis data and three variants identified as statistically significantly associated with change in cholecalciferol levels are presented in Table 2.

Genotypes of statistically significant variants were extracted and assessed for eQTLs with miR-155-5p PBMC expression. We observe statistically significant eQTLs with miR-155-5p only for rs2271367 (F: 6.630; *p* = 0.003), but not for rs74849864 (F: 0.602; *p* = 0.442) and rs62129063 (F: 1.065; *p* = 0.307). Moreover, the recessive model for the G allele of rs2271367 is also proven to be statistically significant for eQTLs with miR-155-5p expression (F: 12.924; *p* = 7 × 10^−4^). 

**Figure 3:**
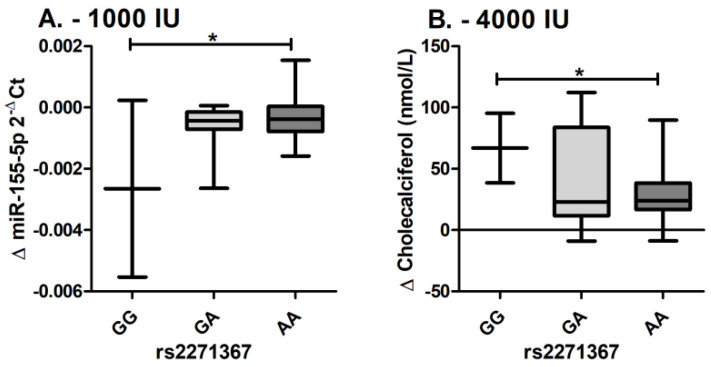
eQTL estimation and delta cholecalciferol levels per rs2271367 genotypes. (**A**) Delta miR-155-5p levels per genotype; (**B**) delta cholecalciferol levels per genotype; * denotes statistically significant difference.

Additionally, it is clearly visible that Δ miR-155-5p levels and Δ cholecalciferol levels are inversely correlated through trends, but a statistically significant correlation is not observed (ρ: 0.171; *p* = 0.172) (Figure 3). It is clearly shown that the maximal decrease in miR-155-5p PBMCs expression is observed within the GG genotype, while the maximal increase in cholecalciferol blood serum levels is also observed within the GG genotype. Furthermore, eQTLs of rs2271367 and the *SARAF* gene are also observed in the GTExPortal database in whole blood (NES: −0.10; *p* = 1.1 × 10^−7^) and sun-exposed skin (NES: −0.20; *p* = 7.0 × 10^−13^) [37].

### 3.4. Target Gene Expression

**Figure 4:**
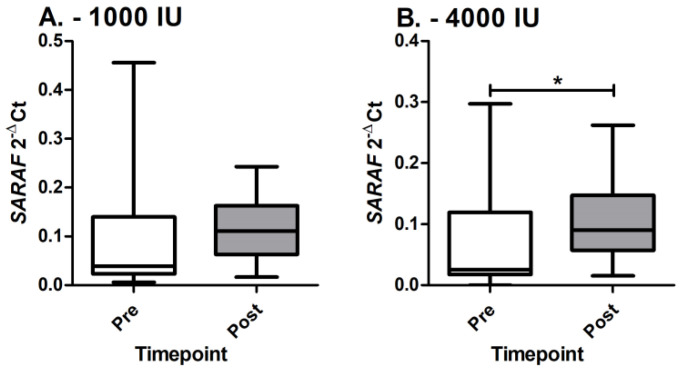
*SARAF* gene expression levels. (**A**) *SARAF* expression levels pre and post in 1000 IU group; (**B**) *SARAF* expression levels pre and post in 4000 IU group; * denotes statistically significant difference.

Based on the aforementioned results, the *SARAF* gene was chosen as the target gene to be validated using the RT-qPCR method. A trend of up-regulation of *SARAF* gene expression is observed in both groups (Figure 4). 

However, statistical significance is observed only in the group receiving 4000 IU (*p* = 0.046), but not in the group receiving 1000 IU (*p* = 0.256). Additionally, no statistically significant differences are observed between groups pre (*p* = 0.163) or post (*p* = 0.378) cholecalciferol supplementation. Subsequently, linear mixed models were also applied in order to assess the *SARAF* gene up-regulation corrected for cholecalciferol dosage, age, sex, and cholecalciferol levels, but, despite the trend, a statistically significant difference between timepoints is not observed (F: 0.995; *p* = 0.321).

## 4. Discussion

In the present study, we assessed the relationship between cholecalciferol supplementation, miR-155-5p expression, miRNA’s target genes, and genetic variants in Slovenian patients with RRMS. The first step consisted of measuring the miR-155-5p expression in patients’ PBMCs before and after supplementation with cholecalciferol. The results show that miRNA expression statistically significantly decreases in both groups and differences in expression between groups is not observed at any point. MiRNAs are short regulatory RNA molecules that play a pivotal role in the modulation of gene expression at the post-transcriptional level [13]. It is also known that a single miRNA molecule can target and change the expression of many genes or possibly many other miRNAs, thus, showing a significant fundamental role in physiological processes [38]. MiR-155 was previously associated with various conditions, autoimmunity, and inflammation states, including multiple sclerosis, neuroinflammation, and other neurological disorders [14,15,16,17,18]. Different mechanisms for miR-155 were proposed in the development of multiple sclerosis. It was suggested that miR-155 promotes blood–brain barrier disruption, promotes demyelination, promotes the development of neuropathic pain, and, thus, leads to neuropsychiatric complications in patients with multiple sclerosis [16]. Previous studies also showed that miR-155 expression was higher in patients with multiple sclerosis in comparison to the controls in peripheral blood leukocytes [20,39]. Furthermore, it is shown that the inhibition of miR-155 expression is effective in preventing processes that are involved in the pathophysiology of multiple sclerosis [16]. A recent study also suggests that miR-155 expression, together with miR-145 expression, in PBMCs is associated with RRMS [22]. However, the later study also demonstrated contradictory findings that higher levels of miR-155 and miR-145 expression was observed in controls and not RRMS patients [22]. Nevertheless, a recent study involving rheumatoid arthritis, also an autoimmune disease, showed that the down-regulation of the molecular axis involving miR-155-5p relieved the disease progression [40], thus, implicating that the decrease in miR-155 plays a crucial role in relieving autoimmune inflammation states.

The second step of the present study is to profile the genome for genetic variants that are associated with cholecalciferol uptake. In order to assess the association of genetic variants with cholecalciferol supplementation, the serum levels of cholecalciferol were assessed a priori. Both groups show a statistically significant increase in cholecalciferol serum levels after supplementation. A between-groups difference is also observed after supplementation, with the group receiving 4000 IU exhibiting significantly higher cholecalciferol serum levels. Subsequently, genome-wide association analysis was performed in order to explore the associations of the variants with Δ cholecalciferol levels and a targeted approach was used in order to identify the variants of interest. The targeted approach was based on the selection of variants harboring near-miRNA-155-5p binding seeds on corresponding target genes extracted from the miRWalk database. We found three statistically significant signals: rs2271367 (*SARAF*), rs74849864 (*TCF4*), and rs62129063 (*SMARCA4*). To the best of our knowledge, none of the aforementioned variants was previously associated with any phenotype or trait.

Additionally, eQTLs of the variants with miR-155-5p were assessed and only for rs2271367 was an eQTL with miRNA observed. An additional eQTL of rs2271367 with the corresponding *SARAF* gene is also evident from the GTExPortal database [37], where eQTLs are listed in whole blood and sun-exposed skin, which indirectly additionally confirms the association of the rs2271367 with cholecalciferol in PBMCs. When assessing the miR-155-5p expression and cholecalciferol serum levels, we observe inverse correlation, but the threshold levels for statistical significance are not met. On the other hand, it is clearly visible that the maximal decrease in miR-155-5p expression is observed within the GG genotype, while the maximal increase in cholecalciferol blood serum levels is also observed within the GG genotype. 

In the third and final step of the present study, we study and evaluate the expression of the *SARAF* gene in PBMCs, which is identified as an miR-155-5p target and is associated with the variant rs2271367 in cholecalciferol association analysis. Both groups show up-regulated *SARAF* gene expression, but statistical significance is observed only for the 4000 IU group. Store-operated calcium entry-associated regulatory factor (*SARAF*) is involved in regulation of store-operated calcium entry and it is located in the endoplasmic reticulum (www.genecards.org, accessed on 26 January 2023). It negatively regulates Ca^2+^ entry involved in protecting cells from Ca^2+^ overfilling, thus, preventing the overload of the cells with excessive Ca^2+^ ions (www.genecards.org, accessed on 26 January 2023). The *SARAF* gene is directly associated with cholecalciferol supplementation since cholecalciferol is responsible for maintaining the extracellular calcium concentrations by controlling the absorption of calcium and by directly exerting effects on bone and parathormone secretion [41]. In response to intracellular Ca^2+^ rise, *SARAF* cooperates with the STIM1 inactivation domain and, subsequently, controls calcium release-activated channels (CRAC) channel Ca^2+^-dependent inactivation [42]. Additionally, *SARAF* is required for proper T-cell-evoked transcription, insinuating that *SARAF* fine-tunes intracellular Ca^2+^ responses and subsequent downstream gene expression in immune cells [42].

Based on the results obtained in the present study, we hypothesize that cholecalciferol supplementation induces up-regulation of the *SARAF* gene through cholecalciferol-maintained calcium concentration, which, in turn, exerts an effect through a possible negative feedback loop between *SARAF* and miR-155-5p expression. Moreover, it is observed that the effect of cholecalciferol supplementation is also genotype-dependent, suggesting that the possible negative feedback loop is variant-driven. 

However, our results are in discrepancy with a previous study that found that *SARAF* may be preferentially expressed in patients with multiple sclerosis [43]. The study showed that *SARAF* induced the expression of pro-inflammatory cytokines, but not anti-inflammatory cytokines [43]. These findings are partially supported with findings that indicate that *SARAF* contributes to T-cell activation through the promotion of TCR-mediated signaling via Ca^2+^–calcineurin–nuclear factor of the activated T-cells (NFAT) pathway [42]. However, it has to be stated that the NFAT pathway is ambiguous. On one hand, NFAT controls the expression of many pro-inflammatory cytokines, but, on the other hand, NFAT has an important role in immune tolerance controlling the differentiation and function of T regulatory and IL-10 producing B regulatory cells, which are required for immune homeostasis and crucial in preventing autoimmunity [44,45,46,47]. The latter findings are in favor of the established hypothesis in the present study. It is noteworthy that in the study where *SARAF* is preferentially expressed in patients with multiple sclerosis, the expression is three-fold higher in patients in comparison to the controls [43], whereas in the present study, we observe the increase in *SARAF* expression at a much lesser magnitude and during the interval between pre- and post-supplementation timepoints. Moreover, we do not observe any statistically significant increase in parathormone (1000 IU: *p* = 0.626 and 4000 IU: *p* = 0.405) or calcium levels (1000 IU: *p* = 0.870 and 4000 IU: *p* = 0.550) in both groups, which indicates that the proposed negative feedback loop between *SARAF* and miR-155-5p expression operates in an environment with maintained homeostasis despite the cholecalciferol supplementation. 

Both studies where discrepancies between miR-155 or *SARAF* expression in regard to the present study were observed were also performed on particular Iranian and Bahraini populations [22,43]. In the later study, the authors also pointed to particular Middle East and Gulf region populations and stated that further studies are required in order to elucidate the role of the *SARAF* gene in multiple sclerosis [43], depicting an awareness of genetic heterogeneity between different populations.

Moreover, it is also shown that 1,25(OH)_2_D_3_ modulates the innate immune axis in mice and suppresses inflammation by down-regulating miR-155 expression [24,48], which, in turn, further supports the connection between cholecalciferol and miR-155 regulation. It is believed that 1,25(OH)_2_D_3_ down-regulates BIC transcription by blocking NF-κB and, thus, decreases the expression of miR-155 [24]. Additionally, in another study significant dysregulation between cholecalciferol serum levels and miR-155/miR-146a was observed in Turkish RRMS patients [49]. Considering the aforementioned statements, the *SARAF* miR-155-5p axis hypothesis might be another mechanism by which cholecalciferol supplementation might decrease miR-155 expression.

The main limitation of the present study is the lack of a control group and, thus, miRNA or *SARAF* expression is not available for a case–control analysis. However, we acknowledge a homogenous Slovenian cohort with RRMS as the strength of our study. Additionally, our analyses were adjusted to confounding environmental variables, such as sun exposure and diet, in order to avoid over-estimation of an effect. 

## 5. Conclusions

In summary, our study used a unique approach to investigate the interplay of cholecalciferol supplementation, miR-155-5p expression, genetic variants, and *SARAF* gene expression. To the best of our knowledge, this is the first time that this continuum of connection was explored and yielded the hypothesis of miRNA-155-5p down-regulation through a proposed negative feedback loop driven by cholecalciferol supplementation via *SARAF* gene expression. The present study firmly warrants further investigations using functional cell models and a large-scale clinical trial in order to elucidate the proposed mechanism.

## Figures and Tables

**Table 1:** Demographics of enrolled patients.

	1000 IU	4000 IU	*p* Value
Sex (M/F)	11/23	11/20	0.800
Age (years)	39.7 ± 9.5	42.2 ± 9.2	0.261
MS duration (months)	9.3 ± 4.7	10.7 ± 6.4	0.485
EDSS	2.0 ± 1.6	2.3 ± 1.4	0.437
MSFC	0.4 ± 0.4	0.2 ± 0.6	0.451
Cholecalciferol	59.3 ± 18.0	56.2 ± 22.0	0.650
Parathyroid hormone	41.8 ± 19.3	46.0 ± 16.8	0.147
Creatinine	62.4 ± 11.9	66.5 ± 14.8	0.269
Calcium	1.2 ± 0.2	1.2 ± 0.2	0.230
Phosphate	1.0 ± 0.2	1.0 ± 0.2	0.916
CRP	3.7 ± 2.2	4.5 ± 3.5	0.137

EDSS: Expanded Disability Status Scale; MSFC: Multiple Sclerosis Functional Composite; CRP: C-reactive protein.

**Table 2:** Statistically significant genomic variants associated with change in cholecalciferol levels located within miR-155-5p binding seeds region.

Variant	Gene	Location	*p* Value
rs2271367	*SARAF*	Chr8:29923732	0.024
rs74849864	*TCF4*	Chr18:52924695	0.022
rs62129063	*SMARCA4*	Chr19:11136006	0.048

## Data Availability

Data available on request due to privacy restrictions. The data presented in this study are available on request from the corresponding author.

## Supplementary Materials

The following supporting information can be downloaded at: https://www.mdpi.com/article/10.3390/genes14061237/s1, Table S1: MiRNA-155-5p selected target genes.

## References

[1] Ramagopalan S.V., Sadovnick A.D. (2011). Epidemiology of multiple sclerosis. Neurol. Clin..

[2] Nylander A., Hafler D.A. (2012). Multiple sclerosis. J. Clin. Investig..

[3] Thompson A.J., Banwell B.L., Barkhof F., Carroll W.M., Coetzee T., Comi G., Correale J., Fazekas F., Filippi M., Freedman M.S. (2018). Diagnosis of multiple sclerosis: 2017 revisions of the McDonald criteria. Lancet Neurol..

[4] Karakatič S., Magdič J., Karakatič S., Omerzu T., Modrič E., Hojs Fabjan T. (2020). Diagnostic relevance of free light chain indices and their relation to the clinical presentation of multiple sclerosis. Acta Med. Biotech..

[5] Popescu B.F., Pirko I., Lucchinetti C.F. (2013). Pathology of multiple sclerosis: Where do we stand?. Continuum.

[6] Mokry L.E., Ross S., Ahmad O.S., Forgetta V., Smith G.D., Goltzman D., Leong A., Greenwood C.M., Thanassoulis G., Richards J.B. (2015). Vitamin D and Risk of Multiple Sclerosis: A Mendelian Randomization Study. PLoS Med..

[7] Salzer J., Hallmans G., Nystrom M., Stenlund H., Wadell G., Sundstrom P. (2012). Vitamin D as a protective factor in multiple sclerosis. Neurology.

[8] Manousaki D., Mitchell R., Dudding T., Haworth S., Harroud A., Forgetta V., Shah R.L., Luan J., Langenberg C., Timpson N.J. (2020). Genome-wide Association Study for Vitamin D Levels Reveals 69 Independent Loci. Am. J. Hum. Genet..

[9] Lucas R.M., Ponsonby A.L., Dear K., Valery P.C., Pender M.P., Taylor B.V., Kilpatrick T.J., Dwyer T., Coulthard A., Chapman C. (2011). Sun exposure and vitamin D are independent risk factors for CNS demyelination. Neurology.

[10] Ramagopalan S.V., Handel A.E., Giovannoni G., Rutherford Siegel S., Ebers G.C., Chaplin G. (2011). Relationship of UV exposure to prevalence of multiple sclerosis in England. Neurology.

[11] Simpson S., Wang W., Otahal P., Blizzard L., van der Mei I.A.F., Taylor B.V. (2019). Latitude continues to be significantly associated with the prevalence of multiple sclerosis: An updated meta-analysis. J. Neurol. Neurosurg. Psychiatry.

[12] Scazzone C., Agnello L., Bivona G., Lo Sasso B., Ciaccio M. (2021). Vitamin D and Genetic Susceptibility to Multiple Sclerosis. Biochem. Genet..

[13] Sondergaard H.B., Hesse D., Krakauer M., Sorensen P.S., Sellebjerg F. (2013). Differential microRNA expression in blood in multiple sclerosis. Mult. Scler..

[14] Mahboobi R., Fallah F., Yadegar A., Dara N., Kazemi Aghdam M., Asgari B., Hakemi-Vala M. (2022). Expression analysis of miRNA-155 level in Helicobacter pylori related inflammation and chronic gastritis. Iran J. Microbiol..

[15] O’Connell R.M., Taganov K.D., Boldin M.P., Cheng G., Baltimore D. (2007). MicroRNA-155 is induced during the macrophage inflammatory response. Proc. Natl. Acad. Sci. USA.

[16] Maciak K., Dziedzic A., Miller E., Saluk-Bijak J. (2021). miR-155 as an Important Regulator of Multiple Sclerosis Pathogenesis. A Review. Int. J. Mol. Sci..

[17] Asadpour-Behzadi A., Kariminik A., Kheirkhah B. (2023). MicroRNA-155 is a main part of proinflammatory puzzle during severe coronavirus disease 2019 (COVID-19). Allergol. Immunopathol..

[18] Rastegar-Moghaddam S.H., Ebrahimzadeh-Bideskan A., Shahba S., Malvandi A.M., Mohammadipour A. (2023). Roles of the miR-155 in Neuroinflammation and Neurological Disorders: A Potent Biological and Therapeutic Target. Cell. Mol. Neurobiol..

[19] McCoy C.E. (2017). miR-155 Dysregulation and Therapeutic Intervention in Multiple Sclerosis. Adv. Exp. Med. Biol..

[20] Paraboschi E.M., Solda G., Gemmati D., Orioli E., Zeri G., Benedetti M.D., Salviati A., Barizzone N., Leone M., Duga S. (2011). Genetic association and altered gene expression of mir-155 in multiple sclerosis patients. Int. J. Mol. Sci..

[21] Lopez-Ramirez M.A., Wu D., Pryce G., Simpson J.E., Reijerkerk A., King-Robson J., Kay O., de Vries H.E., Hirst M.C., Sharrack B. (2014). MicroRNA-155 negatively affects blood-brain barrier function during neuroinflammation. FASEB J..

[22] Ali Ashrafi S., Asadi M., Shanehbandi D., Sadigh Eteghad S., Fazlollahi A., Nejadghaderi S.A., Shaafi S. (2022). Association between miRNA-145 and miRNA-155 expression in peripheral blood mononuclear cells of patients with multiple sclerosis: A case-control study. BMC Neurol..

[23] Keller A., Leidinger P., Lange J., Borries A., Schroers H., Scheffler M., Lenhof H.P., Ruprecht K., Meese E. (2009). Multiple sclerosis: MicroRNA expression profiles accurately differentiate patients with relapsing-remitting disease from healthy controls. PLoS ONE.

[24] Li Y.C., Chen Y., Liu W., Thadhani R. (2014). MicroRNA-mediated mechanism of vitamin D regulation of innate immune response. J. Steroid. Biochem. Mol. Biol..

[25] Hanwell H.E., Vieth R., Cole D.E., Scillitani A., Modoni S., Frusciante V., Ritrovato G., Chiodini I., Minisola S., Carnevale V. (2010). Sun exposure questionnaire predicts circulating 25-hydroxyvitamin D concentrations in Caucasian hospital workers in southern Italy. J. Steroid Biochem. Mol. Biol..

[26] Hedlund L., Brekke H.K., Brembeck P., Augustin H. (2014). A Short Questionnaire for Assessment of Dietary Vitamin D Intake. Eur. J. Nutr. Food Saf..

[27] Livak K.J., Schmittgen T.D. (2001). Analysis of relative gene expression data using real-time quantitative PCR and the 2(-Delta Delta C(T)) Method. Methods.

[28] Bates D., Mächler M., Bolker B., Walker S. (2015). Fitting Linear Mixed-Effects Models Using lme4. J. Stat. Softw..

[29] Anderson C.A., Pettersson F.H., Clarke G.M., Cardon L.R., Morris A.P., Zondervan K.T. (2010). Data quality control in genetic case-control association studies. Nat. Protoc..

[30] Das S., Forer L., Schonherr S., Sidore C., Locke A.E., Kwong A., Vrieze S.I., Chew E.Y., Levy S., McGue M. (2016). Next-generation genotype imputation service and methods. Nat. Genet..

[31] Chang C.C., Chow C.C., Tellier L.C., Vattikuti S., Purcell S.M., Lee J.J. (2015). Second-generation PLINK: Rising to the challenge of larger and richer datasets. Gigascience.

[32] Zhang F., Boerwinkle E., Xiong M. (2014). Epistasis analysis for quantitative traits by functional regression model. Genome Res..

[33] Sticht C., De La Torre C., Parveen A., Gretz N. (2018). miRWalk: An online resource for prediction of microRNA binding sites. PLoS ONE.

[34] Hsu J.B., Chiu C.M., Hsu S.D., Huang W.Y., Chien C.H., Lee T.Y., Huang H.D. (2011). miRTar: An integrated system for identifying miRNA-target interactions in human. BMC Bioinform..

[35] Altschul S.F., Madden T.L., Schaffer A.A., Zhang J., Zhang Z., Miller W., Lipman D.J. (1997). Gapped BLAST and PSI-BLAST: A new generation of protein database search programs. Nucleic Acids Res..

[36] Gorenjak M., Repnik K., Jezernik G., Jurgec S., Skok P., Potocnik U. (2019). Genetic prediction profile for adalimumab response in Slovenian Crohn’s disease patients. Z Gastroenterol..

[37] Consortium G. (2018). Erratum: Genetic effects on gene expression across human tissues. Nature.

[38] Tonacci A., Bagnato G., Pandolfo G., Billeci L., Sansone F., Conte R., Gangemi S. (2019). MicroRNA Cross-Involvement in Autism Spectrum Disorders and Atopic Dermatitis: A Literature Review. J. Clin. Med..

[39] Aljawadi Z.A., Al-Derzi A.R., Abdul-Majeed B.A., Almahdawi A.M. (2016). MicroRNAs (20a, 146a, 155, and 145) expressions in a sample of Iraqi patients with multiple sclerosis. J. Fac. Med. Baghdad.

[40] Hu X., Li M., Zhang Y., Sang K., Li W., Liu B., Wan L., Du B., Qian J., Meng F. (2023). An innovative immunotherapeutic strategy for rheumatoid arthritis: Selectively suppressing angiogenesis and osteoclast differentiation by fully human antibody targeting thymocyte antigen-1. Exp. Cell. Res..

[41] Mithal A., Wahl D.A., Bonjour J.P., Burckhardt P., Dawson-Hughes B., Eisman J.A., El-Hajj Fuleihan G., Josse R.G., Lips P., Morales-Torres J. (2009). Global vitamin D status and determinants of hypovitaminosis D. Osteoporos. Int..

[42] Zomot E., Achildiev Cohen H., Dagan I., Militsin R., Palty R. (2021). Bidirectional regulation of calcium release-activated calcium (CRAC) channel by SARAF. J. Cell. Biol..

[43] Taha S., Aljishi M., Alsharoqi I., Bakhiet M. (2015). Differential upregulation of the hypothetical transmembrane protein 66 (TMEM66) in multiple sclerosis patients with potential inflammatory response. Biomed. Rep..

[44] Macian F. (2005). NFAT proteins: Key regulators of T-cell development and function. Nat. Rev. Immunol..

[45] Bhattacharyya S., Deb J., Patra A.K., Thuy Pham D.A., Chen W., Vaeth M., Berberich-Siebelt F., Klein-Hessling S., Lamperti E.D., Reifenberg K. (2011). NFATc1 affects mouse splenic B cell function by controlling the calcineurin-NFAT signaling network. J. Exp. Med..

[46] Vaeth M., Muller G., Stauss D., Dietz L., Klein-Hessling S., Serfling E., Lipp M., Berberich I., Berberich-Siebelt F. (2014). Follicular regulatory T cells control humoral autoimmunity via NFAT2-regulated CXCR5 expression. J. Exp. Med..

[47] Vaeth M., Feske S. (2018). NFAT control of immune function: New Frontiers for an Abiding Trooper. F1000Research.

[48] Chen Y., Liu W., Sun T., Huang Y., Wang Y., Deb D.K., Yoon D., Kong J., Thadhani R., Li Y.C. (2013). 1,25-Dihydroxyvitamin D promotes negative feedback regulation of TLR signaling via targeting microRNA-155-SOCS1 in macrophages. J. Immunol..

[49] Saridas F., Tezcan Unlu H., Cecener G., Egeli U., Sabour Takanlou M., Sabour Takanlou L., Tunca B., Zarifoglu M., Turan O.F., Taskapilioglu O. (2022). The expression and prognostic value of miR-146a and miR-155 in Turkish patients with multiple sclerosis. Neurol. Res..

