# Supplementary material for: Cholecalciferol Supplementation Induced Up-Regulation of SARAF Gene and Down-Regulated miR-155-5p Expression in Slovenian Patients with Multiple Sclerosis
Source: Genes (Basel). 2023 Jun 8;14(6):1237. doi: 10.3390/genes14061237 (PMC10297972; doi:10.3390/genes14061237)
Supplement: Supplementary file 1 [file genes-14-01237-s001.zip › Supp/genes-2417538_UNMARKED.pdf]

genes

MDPI

Article

Cholecalciferol supplementation induced up-regulation of *SARAF* gene down-regulates miR-155-5p expression in Slovenian patients with multiple sclerosis

Saša Gselman[1], Tanja Hojs Fabjan[1,2], Anja Bizjak [3], Uroš Potočnik[3,4,5]* and Mario Gorenjak[3]*

[1] Clinic of Neurology, University Clinical Centre Maribor, Maribor, Slovenia; sasa.gselman@gmail.com (SG); tanja.hojsfabjan@ukc-mb.si (THF)
[2] Department of Neurology, University of Maribor, Faculty of Medicine, Maribor, Slovenia; tanja.hojsfabjan@ukc-mb.si (THF)
[3] Center for human molecular genetics and pharmacogenomics, University of Maribor, Faculty of Medicine, Maribor, Slovenia; mario.gorenjak@um.si (MG); anja.bizjak2@um.si (AB); uros.potocnik@um.si (UP)
[4] Faculty of chemistry and chemical engineering, University of Maribor, Maribor, Slovenia; uros.potocnik@um.si (UP)
[5] Department for science and research, University Clinical Centre Maribor, Maribor, Slovenia; uros.potocnik@um.si (UP)
* Correspondence: uros.potocnik@um.si; Tel.: +38622345854 (UP); mario.gorenjak@um.si; Tel: +38622345880 (MG). UP and MG contributed equally to this work.

Citation: Gselman, S.; Hojs, T.F.; Bizjak, A.; Potočnik, U.; Gorenjak, M. Cholecalciferol Supplementation Induced Up-Regulation of *SARAF* Gene and Down-Regulated miR-155-5p Expression in Slovenian Patients with Multiple Sclerosis. *Genes* **2023**, *14*, 1237. https://doi.org/10.3390/genes14061237

Academic Editor: Claudia Ricci

Received: 10 May 2023
Revised: 5 June 2023
Accepted: 6 June 2023
Published: 8 June 2023

Copyright: © 2023 by the authors. Licensee MDPI, Basel, Switzerland. This article is an open access article distributed under the terms and conditions of the Creative Commons Attribution (CC BY) license (https://creativecommons.org/licenses/by/4.0/).

Abstract: Multiple sclerosis is a common immune-mediated inflammatory and demyelinating disease. Lower cholecalciferol levels are an established environmental risk factor in multiple sclerosis. Although cholecalciferol supplementation in multiple sclerosis is widely accepted, optimal serum levels are still debated. Moreover, how cholecalciferol affects pathogenic disease mechanisms is still unclear. In the present study we enrolled 65 relapsing-remitting multiple sclerosis patients who were double-blindly divided into two groups with low and high cholecalciferol supplementation, respectively. In addition to clinical and environmental parameters, we obtained peripheral blood mononuclear cells to analyze DNA, RNA, and miRNA molecules. Importantly, we investigated miRNA-155-5p, a previously published pro-inflammatory miRNA in multiple sclerosis known to be correlated to cholecalciferol levels. Our results show decrease of miR-155-5p expression after cholecalciferol supplementation in both dosage groups, consistent with previous observations. Subsequent genotyping, gene expression and eQTL analyses revealed correlations between miR-155-5p and *SARAF* gene, which is playing a role in regulation of calcium release-activated channels. As such, the present study is the first to explore and suggests that the *SARAF* miR-155-5p axis hypothesis might be another mechanism by which cholecalciferol supplementation might decrease miR-155 expression. This association highlights the importance of cholecalciferol supplementation in multiple sclerosis and encourages further investigation and functional cell studies.

Keywords: multiple sclerosis; relapsing-remitting multiple sclerosis; micro RNA; miR-155-5p; *SARAF* gene

1. Introduction

Multiple sclerosis is a common immune-mediated inflammatory and demyelinating disease of the central nervous system, which makes it the leading cause of disability among young adults [1,2]. Diagnosis in patients with suggestive clinical presentation is made using McDonald's criteria of dissemination in space and time [3]. Nevertheless, sometimes there is a necessity for increasing the diagnostic confidence of imaging with oligoclonal bands or free light chains in cerebrospinal fluid [4]. Multiple sclerosis is a

complex disease that results from genetic, epigenetic, and environmental factors, which trigger autoimmune mechanisms, which in turn cause demyelination, subsequent axonal damage, and neurodegeneration [5]. Cholecalciferol, generally known also as vitamin D, is one of the main environmental factors involved in the pathogenesis of multiple sclerosis and it was previously shown that lower cholecalciferol serum levels are associated with an increased risk of developing multiple sclerosis and greater disease activity [6,7]. However, the exact impact of cholecalciferol supplementation on disease activity remains unclear and optimal serum levels and supplementation doses remain controversial.

In the last decade research data is showcasing that cholecalciferol serum levels are associated with genes or genetic loci involved in cholecalciferol metabolism, transport, and elimination [8]. These findings are leading to further research of these genetic loci because of well-known correlations between the disease prevalence and geographic latitude, which can be explained by the effect of ultraviolet radiation or cholecalciferol serum levels [7,9-11]. However, the open question remains if genetic loci associated with cholecalciferol serum levels are also related to disease susceptibility [12].

Additionally, epigenetic factors such as small non-coding microRNA molecules (miRNAs) are dysregulated in many autoimmune diseases including multiple sclerosis [13-18]. They are involved in the post-transcriptional regulation of gene expression[13]. MiR-155 is one of the main pro-inflammatory miRNAs playing role in the pathogenesis of multiple sclerosis [16]. MiR-155 is encoded by the host gene *MIR155HG* [16]. Overexpression of miR-155 has been noticed in active brain lesions and also in peripheral blood cells [16,19]. The exerted pro-inflammatory effect of miR-155 has an impact on the infiltration of peripheral immune cells, causes demyelination via microglia activation, phagocytosis of myelin by macrophages, differentiation of T-cells and also contributes to increased permeability of the blood-brain barrier [19-21].

The roles of miRNA molecules in multiple sclerosis are flagged and proposed as promising diagnostic biomarkers [13,22]. Furthermore, it was shown that miRNAs can accurately differentiate patients with relapsing-remitting multiple sclerosis (RRMS) from healthy controls [23]. Additionally, it was also shown that the biological active form of vitamin D (1,25$(OH)_2D_3$) modulates and suppresses inflammation by down-regulating miR-155 expression [24], which in turn establishes a bridge between miR-155 and cholecalciferol. Nevertheless, studied and established associations between co-regulation of genetic, epigenetic (miRNAs), and cholecalciferol supplementation are scarce.

Therefore, in order to investigate the possible interplay between cholecalciferol, genetic and epigenetic factors,we conducted a double-blind randomized study during winter time on a specific cohort of Slovenian relapsing-remitting multiple sclerosis (RRMS) patients with the aim to explore the continuum of connections between miR-155-5p expression, cholecalciferol supplementation, genetic variants, and miR-155-5p genetic targets as follows:

1. Measure the miR-155-5p expression in peripheral blood mononuclear cells (PBMCs);
2. Profile the genome for variants associated with cholecalciferol uptake;
3. Stringently select genetic targets of miR-155-5p and extract genetic variants associated with cholecalciferol pathways ;
4. Calculate and assess expression quantitative trait loci (eQTLs) between aforementioned genetic variants, miR-155-5p, and miR-155-5p target genes;
5. Identify target genes where both eQTLs are observed and measure the expression of the corresponding gene.

2. Materials and Methods

2.1. Subjects

We enrolled 65 patients with diagnosed relapsing-remitting multiple sclerosis from the Department of Neurologic Diseases University Clinical Centre Maribor, Slovenia. The

patients were double blindly divided into two groups for low dose (1000 IU per day) and high dose (4000 IU per day) of cholecalciferol supplementation. All patients were aged 18 - 60 years, were on immunomodulatory therapy, and had Expanded Disability Status Scale (EDSS) <5. The demographic data before supplementation is summarized in table 1. Exclusion criteria were taking cholecalciferol supplementation during three month wash-out period before the study, pregnancy or breastfeeding, relapse of the disease or corticosteroid treatment in the last month, any viral or inflammatory disease at the enrollment, renal dysfunction, high calcium or parathyroid hormone, change of the immunomodulatory therapy in the last three months before enrollment, concomitant autoimmune diseases and anamnesis of hyperparathyroidism, liver disease, tuberculosis, sarcoidosis or kidney stones. The patients were receiving oil suspension of cholecalciferol (Fresuvit D_3, Fresenius Kabi Austria GmbH) during four winter months from November until February. Before and after cholecalciferol supplementation EDSS and the Multiple Sclerosis Functional Composite (MSFC) score were assessed. Sun Exposure Questionnaire [25] and Short Questionnaire for Assessment of Dietary Vitamin D Intake [26] diaries were filled out during the study.

Table 1. Demographics of enrolled patients.

	1000 IU	4000 IU	P value
Sex (M/F)	11/23	11/20	0.800
Age (years)	39.7±9.5	42.2±9.2	0.261
MS duration (months)	9.3±4.7	10.7±6.4	0.485
EDSS	2.0±1.6	2.3±1.4	0.437
MSFC	0.4±0.4	0.2±0.6	0.451
Cholecalciferol	59.3±18.0	56.2±22.0	0.650
Parathyroid hormone	41.8±19.3	46.0±16.8	0.147
Creatinine	62.4±11.9	66.5±14.8	0.269
Calcium	1.2±0.2	1.2±0.2	0.230
Phosphate	1.0±0.2	1.0±0.2	0.916
CRP	3.7±2.2	4.5±3.5	0.137

EDSS: Expanded Disability Status Scale; MSFC: Multiple Sclerosis Functional Composite; CRP: C-reactive protein.

2.2. *Sample collection*

At the time of enrollment and at the end of the four-month period we collected 12 mL peripheral venous blood into potassium salt of Ethylene Diamine Tetra Acetic acid (K_2EDTA) tubes for mononuclear cell isolation and 3×6 mL into serum tubes for blood biochemistry. Measured biochemistry parameters were serum cholecalciferol level using Cobas e601 apparatus (Roche Diagnostics, Penzberg, Germany), creatinine, calcium, phosphate, C-reactive protein using Siemens Dimesion Vista apparatus(Siemens HealthCare Diagnostics, Newark, DE, USA) and parathyroid hormone using Cobas e411 apparatus (Roche).

2.3. *DNA, mRNA, and miRNA extraction*

DNA and mRNA were extracted from one portion of PBMCs using TRI-reagent (Merck,Darmstadt, Germany) according to the manufacturer's instructions. MiRNA was extracted and purified from the other portion of PBMCs using miRNeasy Mini Kit (Qiagen, Germantown, Maryland, USA) according to the manufacturer's instructions. Purity and concentration of nucleic acids were assessed using Synergy 2 spectrophotometer (Biotek, Winooski, VT, USA) and Qubit (Thermo Fisher, Waltham, MA, USA).

2.4. MiR-155-5p RT-qPCR

MiRNA RT-qPCR was performed using miRCURY LNA RT Kit, miRCURY LNA SYBR Green PCR Kit, and miRCURY LNA miRNA PCR Assay (Qiagen) according to the manufacturer's instructions. PCR Assays used were miR-155-5p as the target miRNA and SNORD49A and SNORD38B as reference miRNAs. Target miRNA was normalized to the geometric mean of reference miRNAs and expressed in linear form of $2^{-\Delta Ct}$ [27]. Statistical analysis of miR-155-5p before and after cholecalciferol supplementation and between groups was carried out using Wilcoxon paired samples rank test and Mann-Whitney U-Test. Additionally, Linear Mixed Models using lme4 R package [28] were performed and fitted using the blocking technique, where a blocking factor was set as a random variable and time point, cholecalciferol dosage, age, sex, and cholecalciferol levels were used as covariates to correct for.

2.5. Genotyping, imputation, and association analysis

DNA was genotyped using genotyping microarray Infinium Global Screening Array (GSA_24v3) and iScan apparatus (Illumina, San Diego, California, USA) according to manufacturer's instructions. Quality control of raw genotype data was performed as previously described [29]. Genotype imputation was carried out using the Michigan Imputation Server Minimac3 genotype imputation algorithm and using the Haplotype Reference Consortium (HRC r1.1 2016) reference panel and SHAPEIT v2.r790 phasing [30]. Association analysis was performed with change (Δ) in cholecalciferol values (post minus pre) as an outcome variable using linear regression implemented in PLINK 2.0 (www.cog-genomics.org/plink/2.0/) [31]. To account for the non-normally distributed outcome variable, a 2-step inverse normal transformation was performed using FRGEpistasis R package [32]. Regression was corrected for sex, age, sun exposure, cholecalciferol dosage, and first four principal components, and was performed using imputed allelic dosages.

2.6. Integration of genomics to miRNA-155-5p targets

Variants identified in association analysis were integrated with miRNA-155-5p target genes, which were listed in miRWalk database (http://mirwalk.umm.uni-heidelberg.de/). Only target genes with a miRNA-gene binding probability of 1.00 and with additional experimental MiRTarBase validation were selected [33,34]. Binding site nucleotide sequence seeds for miRNA on selected target genes were identified on GRCh37 DNA nucleotide sequences using BLAST (https://blast.ncbi.nlm.nih.gov/Blast.cgi) [35]. Genomic variants ±200 bp (10times the average of the binding region length in order to expand the search region) from miRNA binding seeds were extracted from association analysis and further analyzed. Evidence of statistically significant signal was considered for variants with adjusted p-value <0.05. Genotypes of statistically significant variants were extracted and assessed for eQTL with miR-155-5p expression using linear mixed models using lme4 R package [28] and fitted using blocking technique where blocking factor was set as a random variable and time point, sex, age, dosage, and genotype were used as covariates to correct for. For variants with significant eQTL with miR-155-5p, additional dominant and recessive models were tested. A statistical significant signal was considered at p-value <0.05.

2.7. RT-qPCR target gene validation

MiR-155-5p targets which were identified in miRNA-genomic integration were validated using the reverse-transcription quantitative polymerase chain reaction (RT-qPCR) method. A total of 1 μg of extracted mRNA was transcribed into cDNA using a high-capacity cDNA reverse transcription kit (Thermo Fisher, Waltham, MA, USA).

Nucleotide sequences for mRNA of target gene *SARAF* (NM_016127.6) were retrieved from the NCBI Nucleotide database (https://www.ncbi.nlm.nih.gov/nuccore/) and primers were designed using IDT OligoAnalyzer Tool (eu.idtdna.com/calc/analyzer). Nucleotide sequences for the *SARAF* gene were as follows: FW 5′- GTTTTGGCAGTGCTTTTACA - 3′ and RV 5′ - ACGAGTCTGAGAAGGGTGTT - 3′. Primers were synthesized by Sigma (Merck, Darmstadt, Germany). Primers for the reference genes *ACTB* and *B2M* were obtained from a previous study [36]. RT-qPCR assays were carried out using Lightcycler 480 SYBR Green I Master Mix and Lightcycler 480 real-time thermocycler (Roche, Basel, Switzerland) according to manufacturer's instructions. 2 μL of 20-fold diluted cDNA (2.5 ng/μL) was used as a template for a single PCR reaction. Melting curves of each sample were analyzed after each run in order to confirm amplification specificity. Raw Ct values were obtained from independent technical duplicates for each sample and normalization of raw Ct values was carried out using the geometric mean of both reference genes and linear expression was calculated using $2^{-\Delta Ct}$ calculation [27] in order to allow statistical analyses. Statistical analysis of *SARAF* expression before and after cholecalciferol supplementation and between groups was carried out using Wilcoxon paired samples rank test and Mann-Whitney U-Test. Additionally, Linear Mixed Models using the lme4 R package [28] were performed and fitted using the blocking technique as aforementioned.

2.8 Statistical analyses

Data were analyzed using R 4.1.3 environment (R Core Team 2020, Vienna, Austria). Statistical differences between nominal categorical variables were estimated using Fisher's exact test. All continuous variables wefirst assessed for normality of distribution using the Kolmogorov-Smirnov test of normality. Statistical differences of continuous variables between two groups were assessed using Mann-Whitney U-Tests. Differences between two timepoints were assessed using Wilcoxon signed rank test. Correlations were estimated using Spearman rank correlations. The dosage effect of cholecalciferol supplementation was estimated using Generalized Linear Models with Δ cholecalciferol values (post minus pre) as an outcome variable corrected for sex, age, and consumption of fish, milk, yogurt, margarine, and sun exposure.

3. Results

3.1. Estimation of cholecalciferol supplementation

First, we assessed the effect of cholecalciferol supplementation on blood serum levels. In both groups receiving 1000 IU and 4000 IU of cholecalciferol supplementation, we observed a statistically significant increase of cholecalciferol in serum (Figure 1A and 1B). In the group receiving 1000 IU the rise in serum levels was from 59.3±18 to 72.5±16.2 nmol/L ($p=3.6\times10^{-5}$) and in the group receiving 4000 IU the rise in serum levels was from 56.2±22 to 106.5±32 nmol/L ($p=1\times10^{-6}$). Between groups difference was also observed after supplementation ($p=1.2\times10^{-5}$). Additionally fitted generalized linear models have also shown a statistically significant effect of the dosage on blood serum cholecalciferol levels (β: 39.98; $P=5.9\times10^{-4}$).

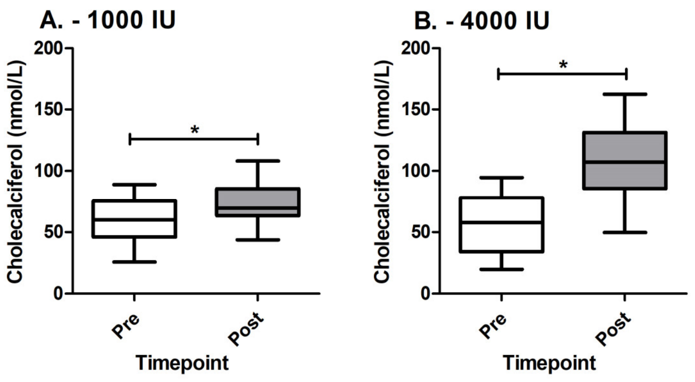

Figure 1. Cholecalciferol levels between groups before and after supplementation. (**a**) Cholecalciferol levels pre and post in 1000 IU group; (**b**) Cholecalciferol levels pre and post in 4000 IU group.

3.2. MiR-155-5p expression

We observed that miR-155-5p expression in PBMCs statistically significantly decreased in both groups receiving 1000 IU and 4000 IU of cholecalciferol supplementation (Figure 2). In the group receiving 1000 IU of cholecalciferol supplementation, the miR-155-5p expression decreased from 0.0015±0.0015 to 0.0008±0.0004 ($P=1.53\times10^{-4}$). In the group receiving 4000 IU of cholecalciferol supplementation the miR-155-5p expression decreased from 0.0013±0.0011 to 0.0009±0.0004 (P=0.021). Additionally, linear mixed models were applied in order to confirm the decrease between timepoints with correction for cholecalciferol dosage, age, sex, and cholecalciferol levels. We again observed that miR-155-5p levels statistically significantly decreased (F: 9.541; P=0.0027). However, between groups difference was not observed after supplementation (p=0.372).

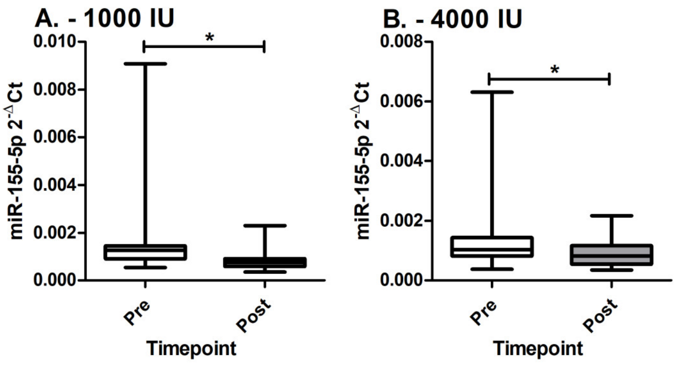

Figure 2. MiR-155-5p expression levels between groups before and after supplementation. (**a**) MiR-155-5p expression levels pre and post in 1000 IU group; (**b**) MiR-155-5p expression levels pre and post in 4000 IU group.

3.3. MiR-155-5p targets, integration to genomics and eQTL estimation

70 miR-155-5p targets were chosen from the miRWalk database and GRCh37 DNA nucleotide sequence seed correspondence was obtained (Table S1). Genomic variants in genomic regions ranging ±200 bp from validated miRNA binding seeds were extracted from association analysis data and three variants identified as statistically significantly associated with change in cholecalciferol levels are presented in Table 2.

Table 2. Statistically significant genomic variants associated with change in cholecalciferol levels located within miR-155-5p binding seeds region.

Variant	Gene	Location	P value
rs2271367	*SARAF*	Chr8:29923732	0.024
rs74849864	*TCF4*	Chr18:52924695	0.022
rs62129063	*SMARCA4*	Chr19:11136006	0.048

Genotypes of statistically significant variants were extracted and assessed for eQTL with miR-155-5p PBMC expression. We observed statistically significant eQTL with miR-155-5p only for rs2271367 (F: 6.630; P=0.003), but not for rs74849864 (F: 0.602; P=0.442) and rs62129063 (F: 1.065; P=0.307). Moreover, recessive model for the G allele of rs2271367 was also proven to be in statistically significant eQTL with miR-155-5p expression (F: 12.924; P=7×10^{-4}).

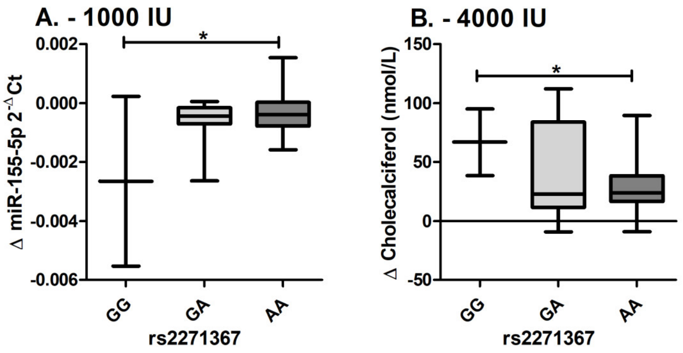

Figure 3. eQTL estimation and delta cholecalciferol levels per rs2271367 genotypes. (**a**) Delta miR-155-5p levels per genotype; (**b**) Delta cholecalciferol levels per genotype.

Additionally, it is clearly visible that Δ miR-155-5p levels and Δ cholecalciferol levels are inversely correlated through trends, but a statistically significant correlation couldn't be observed (ϱ: 0.171; P=0.172) (Figure 3). It is clearly shown that the maximal decrease in miR-155-5p PBMCs expression was observed within GG genotype, while the maximal increase in cholecalciferol blood serum levels was also observed within GG genotype. Furthermore, eQTL of rs2271367 and *SARAF* gene was also observed in GTExPortal database in whole blood (NES:-0.10; P=1.1×10^{-7}) and sun-exposed skin (NES:-0.20; P=7.0×10^{-13}) [37].

3.4. Target gene expression

Based on the aforementioned results,the *SARAF* gene was chosen as the target gene to be validated using the RT-qPCR method. Trend of up-regulation of *SARAF* gene expression was observed in both groups.

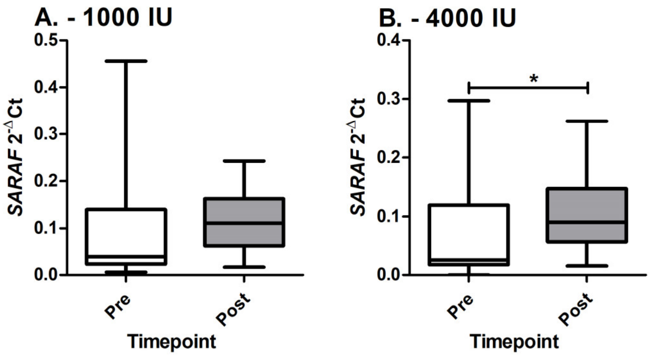

Figure 4. *SARAF* gene expression levels. (**a**) *SARAF* expression levels pre and post in 1000 IU group; (**b**) *SARAF* expression levels pre and post in 4000 IU group.

However, statistical significance was observed only in the group receiving 4000 IU (P=0.046), but not in the group receiving 1000 IU (P=0.256).Additionally, no statistically significant differences were observed between groups pre (P=0.163) or post (P=0.378) cholecalciferol supplementation. Subsequently, linear mixed models were also applied in order to assess the *SARAF* gene up-regulation corrected for cholecalciferol dosage, age, sex, and cholecalciferol levels, but despite the trend statistically significant difference between timepoints was not observed (F: 0.995; P=0.321).

4. Discussion

In the present study, we assessed the relationship between cholecalciferol supplementation, miR-155-5p expression, miRNA's target genes, and genetic variants in Slovenian patients with RRMS. The first step consisted of measuring the miR-155-5p expression in patients' PBMCs before and after supplementation with cholecalciferol. The results have shown that miRNA expression statistically significantly decreased in both groups and differences in expression between groups were not observed at any point. MiRNAs are short regulatory RNA molecules,which are playing a pivotal role in the modulation of gene expression at the post-transcriptional level [13]. It is also known that a single miRNA molecule can target and change the expression of many genes or possibly many other miRNAs and thus, showing a significant fundamental role in physiological processes [38]. MiR-155 was previously associated with various conditions, autoimmunity, and inflammation states, including multiple sclerosis, neuroinflammation, and other neurological disorders [14-18]. Different mechanisms for miR-155 were proposed in the development of multiple sclerosis. It was suggested that miR-155 promotes blood-brain barrier disruption, promotes demyelination, promotes the development of neuropathic pain, and thus leads to neuropsychiatric complications in patients with multiple sclerosis [16]. Previous studies have also shown that miR-155 expression was higher in patients with multiple sclerosis in comparison to the controls in peripheral blood leukocytes [20,39]. Furthermore, it was shown that inhibition of miR-155 expression is effective in preventing processes, which are involved in the pathophysiology of multiple sclerosis [16]. A recent study also suggested that miR-155 expression together with miR-145 expression in PBMCs is associated with RRMS [22]. However, the later study has also demonstrated contradictory findings that higher levels of miR-155 and miR-145 expression was observed in controls and not RRMS patients [22]. Nevertheless, a recent study involving rheumatoid arthritis, also an autoimmune disease, has shown that down-regulation of molecular axis involving miR-155-5p relieved the disease progres-

sion [40], thus implicating that the decrease of miR-155 plays a crucial role in relieving autoimmune inflammation states.

The second step of the present study was to profile the genome for genetic variants which are associated with cholecalciferol uptake. In order to assess the association of genetic variants with cholecalciferol supplementation, the serum levels of cholecalciferol were *a priori* assessed. Both groups have shown a statistically significant increase in cholecalciferol serum levels after supplementation. Between groups difference was also observed after supplementation where the group receiving 4000 IU exhibited significantly higher cholecalciferol serum levels. Subsequently, genome-wide association analysis was performed in order to explore the associations of the variants with Δ cholecalciferol levels and targeted approach was used in order to identify the variants of interest. Targeted approach was based on the selection of variants harboring near miRNA-155-5p binding seeds on corresponding target genes extracted from miRWalk database. We found three statistically significant signals: rs2271367 (*SARAF*), rs74849864 (*TCF4*), and rs62129063 (*SMARCA4*). To the best of our knowledge, none of the aforementioned variants was previously associated with any phenotype or trait.

Additionally, eQTLs of the variants with miR-155-5p were assessed and only for rs2271367 an eQTL with miRNA was observed. Additional eQTL of rs2271367 with corresponding *SARAF* gene is also evident from GTExPortal database [37] where eQTLs are listed in whole blood and sun-exposed skin, which indirectly additionally confirms the association of the rs2271367 with cholecalciferol in PBMCs. When assessing the miR-155-5p expression and cholecalciferol serum levels, we observed inverse correlation, but the threshold levels for statistical significance were not met, but on the other hand, it is clearly visible that the maximal decrease in miR-155-5p expression was observed within GG genotype, while the maximal increase in cholecalciferol blood serum levels was also observed within GG genotype.

In the third and final step of the present study, we studied and evaluated the expression of the *SARAF* gene in PBMCs, which was identified as miR-155-5p target and is associated with the variant rs2271367 in cholecalciferol association analysis. Both groups have shown up-regulated *SARAF* gene expression, but statistical significance was observed only for the 4000 IU group. Store-operated calcium entry associated regulatory factor (*SARAF*) is involved in regulation of store-operated calcium entry and it is located in the endoplasmic reticulum (www.genecards.org). It negatively regulates Ca^{2+} entry involved in protecting cells from Ca^{2+} overfilling thus preventing the overload of the cells with excessive Ca^{2+} ions (www.genecards.org). *SARAF* gene is directly associated to cholecalciferol supplementation since cholecalciferol is responsible for maintaining the extracellular calcium concentrations by controlling the absorption of calcium and by directly exerting effects on bone and parathormone secretion [41]. In response to intracellular Ca^{2+} rise, *SARAF* cooperates with STIM1 inactivation domain and subsequently controls calcium release-activated channels (CRAC) channel Ca^{2+}-dependent inactivation [42]. Additionally, *SARAF* is required for proper T-cell-evoked transcription insinuating that *SARAF* fine-tunes intracellular Ca^{2+} responses and subsequent downstream gene expression in immune cells [42].

Based on the results obtained in the present study we hypothesize that cholecalciferol supplementation induces up-regulation of the *SARAF* gene through cholecalciferol-maintained calcium concentration, which in turn exerts an effect through a possible negative feedback loop between *SARAF* and miR-155-5p expression. Moreover, it was observed that the effect of cholecalciferol supplementation is also genotype dependent, suggesting the possible negative feedback loop is variant driven.

However, our results are in discrepancy with a previous study which found that *SARAF* may be preferentially expressed in patients with multiple sclerosis [43]. The study has shown that *SARAF* induced the expression of pro-inflammatory cytokines, but not anti-inflammatory cytokines [43]. These findings are partially supported with findings, which indicate that *SARAF* contributes to T-cell activation through the promotion of

TCR-mediated signaling via Ca^{2+}-calcineurin-nuclear factor of activated T-cells (NFAT) pathway [42]. However, it has to be stated that the NFAT pathway is ambiguous. On one hand, NFAT controls the expression of many pro-inflammatory cytokines, but on the other hand, NFAT has an important role in immune tolerance controlling the differentiation and function of T regulatory and IL-10 producing B regulatory cells, which are required for immune homeostasis and crucial in preventing autoimmunity [44-47]. The latter findings are in favor of the established hypothesis in the present study. Noteworthy, in the study where *SARAF* was preferentially expressed in patients with multiple sclerosis, the expression was 3-fold higher in patients in comparison to the controls [43], whereas in the present study, we observed the increase of *SARAF* expression at a much lesser magnitude and on the interval between pre- and post-supplementation timepoints. Moreover, we didn't observe any statistically significant increase in parathormone or calcium levels in both groups (data not shown), which indicates that the proposed negative feedback loop between *SARAF* and miR-155-5p expression operates in an environment with maintained homeostasis despite the cholecalciferol supplementation.

Both studies where discrepancies between miR-155 or *SARAF* expression in regard to the present study were observed were also performed on particular Iranian and Bahrain populations [22,43]. In the later study authors also pointed to particular Middle East and Gulf region populations and stated that further studies are required in order to elucidate the role of the *SARAF* gene in multiple sclerosis [43], depicting awareness of genetic heterogeneity between different populations.

Moreover, it was also shown that $1,25(OH)_2D_3$ modulates innate immune axis in mice and suppresses inflammation by down-regulating miR-155 expression [24,48], which in turn further supports the connection between cholecalciferol and miR-155 regulation. It is believed that $1,25(OH)_2D_3$ down-regulates *bic* transcription by blocking NF-κB and thus, decreases the expression of miR-155 [24]. Additionally, in another study significant dysregulation between cholecalciferol serum levels and miR-155/miR-146a was observed in RRMS Turkish patients [49]. Considering the aforementioned statements, the *SARAF* miR-155-5p axis hypothesis might be another mechanism by which cholecalciferol supplementation might decrease miR-155 expression.

The main limitation of the present study is the lack of a control group and thus, miRNA or *SARAF* expression was not available for a case-control analysis. However, we acknowledge a homogenous Slovenian cohort with RRMS as the strength of our study. Additionally, our analyses were adjusted to environmental confounding variables such as sun exposure and diet in order to avoid over-estimation of an effect.

5. Conclusions

In summary, our study used a unique approach to investigate the interplay of cholecalciferol supplementation, miR-155-5p expression, genetic variants, and *SARAF* gene expression. To the best of our knowledge, this is the first time that this continuum of connection was explored and yielded the hypothesis of miRNA-155-5p down-regulation through a proposed negative feedback loop driven by cholecalciferol supplementation via *SARAF* gene expression. The present study firmly warrants further investigations using functional cell models and on a larger-scale clinical trial in order to elucidate the proposed mechanism.

Author Contributions: Conceptualization, M.G., S.G., T.H.F. and U.P.; methodology, M.G. and S.G.; validation, M.G., S.G., U.P. and T.H.F.; formal analysis, M.G., S.G., A.B.; investigation, M.G., S.G. and A.B.; resources, S.G., T.H.F. and U.P.; data curation, M.G., S.G. and A.B.; writing—original draft preparation, M.G., S.G. and A.B.; writing—review and editing, M.G., S.G., U.P. and T.H.F.; visualization, M.G., S.G.; supervision, M.G., U.P. and T.H.F.; project administration, S.G.; funding acquisition, S.G., U.P. and T.H.F. All authors have read and agreed to the published version of the manuscript.

Funding: This research was funded by University Medical Centre Maribor (internal research program), grant number IRP-2018/01-08. This research was also funded by the Slovenian Research Agency Research Core Funding P3-0427 and research grant no. J3-4998.

Institutional Review Board Statement: The study was conducted in accordance with the Declaration of Helsinki, and approved by the Ethics Committee of Slovenian National Committee for Medical Ethics (0120-580/2017/4).

Informed Consent Statement: Informed consent was obtained from all subjects involved in the study.

Data Availability Statement: Data available on request due to privacy restrictions. The data presented in this study are available on request from the corresponding author.

Acknowledgments: The authors would like to thank the patients for participation in this study.

Conflicts of Interest: The authors declare no conflict of interest. The funders had no role in the design of the study; in the collection, analyses, or interpretation of data; in the writing of the manuscript; or in the decision to publish the results.

References

1. Ramagopalan, S.V.; Sadovnick, A.D. Epidemiology of multiple sclerosis. *Neurol Clin* **2011**, *29*, 207-217, doi:10.1016/j.ncl.2010.12.010
S0733-8619(10)00163-5 [pii].
2. Nylander, A.; Hafler, D.A. Multiple sclerosis. *J Clin Invest* **2012**, *122*, 1180-1188, doi:58649 [pii]
10.1172/JCI58649.
3. Thompson, A.J.; Banwell, B.L.; Barkhof, F.; Carroll, W.M.; Coetzee, T.; Comi, G.; Correale, J.; Fazekas, F.; Filippi, M.; Freedman, M.S.; et al. Diagnosis of multiple sclerosis: 2017 revisions of the McDonald criteria. *Lancet Neurol* **2018**, *17*, 162-173, doi:S1474-4422(17)30470-2 [pii]
10.1016/S1474-4422(17)30470-2.
4. Karakatič, S.; Magdič, J.; Karakatič, S.; Omerzu, T.; Modrič, E.; Hojs Fabjan, T. Diagnostic relevance of free light chain indices and their relation to the clinical presentation of multiple sclerosis. *Acta Medico-Biotechnica* **2020**, *13*, 23-32.
5. Popescu, B.F.; Pirko, I.; Lucchinetti, C.F. Pathology of multiple sclerosis: where do we stand? *Continuum (Minneap Minn)* **2013**, *19*, 901-921, doi:10.1212/01.CON.0000433291.23091.65
00132979-201308000-00009 [pii]
CON19406 [pii].
6. Mokry, L.E.; Ross, S.; Ahmad, O.S.; Forgetta, V.; Smith, G.D.; Goltzman, D.; Leong, A.; Greenwood, C.M.; Thanassoulis, G.; Richards, J.B. Vitamin D and Risk of Multiple Sclerosis: A Mendelian Randomization Study. *PLoS Med* **2015**, *12*, e1001866, doi:10.1371/journal.pmed.1001866
e1001866
PMEDICINE-D-15-00749 [pii].
7. Salzer, J.; Hallmans, G.; Nystrom, M.; Stenlund, H.; Wadell, G.; Sundstrom, P. Vitamin D as a protective factor in multiple sclerosis. *Neurology* **2012**, *79*, 2140-2145, doi:10.1212/WNL.0b013e3182752ea8
79/21/2140 [pii].
8. Manousaki, D.; Mitchell, R.; Dudding, T.; Haworth, S.; Harroud, A.; Forgetta, V.; Shah, R.L.; Luan, J.; Langenberg, C.; Timpson, N.J.; et al. Genome-wide Association Study for Vitamin D Levels Reveals 69 Independent Loci. *Am J Hum Genet* **2020**, *106*, 327-337, doi:S0002-9297(20)30017-3 [pii]
10.1016/j.ajhg.2020.01.017.
9. Lucas, R.M.; Ponsonby, A.L.; Dear, K.; Valery, P.C.; Pender, M.P.; Taylor, B.V.; Kilpatrick, T.J.; Dwyer, T.; Coulthard, A.; Chapman, C.; et al. Sun exposure and vitamin D are independent risk factors for CNS demyelination. *Neurology* **2011**, *76*, 540-548, doi:10.1212/WNL.0b013e31820af93d
76/6/540 [pii].
10. Ramagopalan, S.V.; Handel, A.E.; Giovannoni, G.; Rutherford Siegel, S.; Ebers, G.C.; Chaplin, G. Relationship of UV exposure to prevalence of multiple sclerosis in England. *Neurology* **2011**, *76*, 1410-1414, doi:10.1212/WNL.0b013e318216715e
76/16/1410 [pii]
WNL202488 [pii].

11. Simpson, S., Jr.; Wang, W.; Otahal, P.; Blizzard, L.; van der Mei, I.A.F.; Taylor, B.V. Latitude continues to be significantly associated with the prevalence of multiple sclerosis: an updated meta-analysis. *J Neurol Neurosurg Psychiatry* **2019**, *90*, 1193-1200, doi:10.1136/jnnp-2018-320189
jnnp-2018-320189 [pii].
12. Scazzone, C.; Agnello, L.; Bivona, G.; Lo Sasso, B.; Ciaccio, M. Vitamin D and Genetic Susceptibility to Multiple Sclerosis. *Biochem Genet* **2021**, *59*, 1-30, doi:10.1007/s10528-020-10010-1
10.1007/s10528-020-10010-1 [pii].
13. Sondergaard, H.B.; Hesse, D.; Krakauer, M.; Sorensen, P.S.; Sellebjerg, F. Differential microRNA expression in blood in multiple sclerosis. *Mult Scler* **2013**, *19*, 1849-1857, doi:10.1177/1352458513490542
1352458513490542 [pii].
14. Mahboobi, R.; Fallah, F.; Yadegar, A.; Dara, N.; Kazemi Aghdam, M.; Asgari, B.; Hakemi-Vala, M. Expression analysis of miRNA-155 level in Helicobacter pylori related inflammation and chronic gastritis. *Iran J Microbiol* **2022**, *14*, 495-502, doi:10.18502/ijm.v14i4.10235
IJM-14-495 [pii].
15. O'Connell, R.M.; Taganov, K.D.; Boldin, M.P.; Cheng, G.; Baltimore, D. MicroRNA-155 is induced during the macrophage inflammatory response. *Proc Natl Acad Sci U S A* **2007**, *104*, 1604-1609, doi:0610731104 [pii]
4915 [pii]
10.1073/pnas.0610731104.
16. Maciak, K.; Dziedzic, A.; Miller, E.; Saluk-Bijak, J. miR-155 as an Important Regulator of Multiple Sclerosis Pathogenesis. A Review. *Int J Mol Sci* **2021**, *22*, doi:10.3390/ijms22094332
4332
ijms22094332 [pii]
ijms-22-04332 [pii].
17. Asadpour-Behzadi, A.; Kariminik, A.; Kheirkhah, B. MicroRNA-155 is a main part of proinflammatory puzzle during severe coronavirus disease 2019 (COVID-19). *Allergol Immunopathol (Madr)* **2023**, *51*, 115-119, doi:10.15586/aei.v51i2.698.
18. Rastegar-Moghaddam, S.H.; Ebrahimzadeh-Bideskan, A.; Shahba, S.; Malvandi, A.M.; Mohammadipour, A. Roles of the miR-155 in Neuroinflammation and Neurological Disorders: A Potent Biological and Therapeutic Target. *Cell Mol Neurobiol* **2023**, *43*, 455-467, doi:10.1007/s10571-022-01200-z
10.1007/s10571-022-01200-z [pii].
19. McCoy, C.E. miR-155 Dysregulation and Therapeutic Intervention in Multiple Sclerosis. *Adv Exp Med Biol* **2017**, *1024*, 111-131, doi:10.1007/978-981-10-5987-2_5.
20. Paraboschi, E.M.; Solda, G.; Gemmati, D.; Orioli, E.; Zeri, G.; Benedetti, M.D.; Salviati, A.; Barizzone, N.; Leone, M.; Duga, S.; et al. Genetic association and altered gene expression of mir-155 in multiple sclerosis patients. *Int J Mol Sci* **2011**, *12*, 8695-8712, doi:10.3390/ijms12128695
ijms12128695 [pii]
ijms-12-08695 [pii].
21. Lopez-Ramirez, M.A.; Wu, D.; Pryce, G.; Simpson, J.E.; Reijerkerk, A.; King-Robson, J.; Kay, O.; de Vries, H.E.; Hirst, M.C.; Sharrack, B.; et al. MicroRNA-155 negatively affects blood-brain barrier function during neuroinflammation. *FASEB J* **2014**, *28*, 2551-2565, doi:10.1096/fj.13-248880
fj.13-248880 [pii].
22. Ali Ashrafi, S.; Asadi, M.; Shanehbandi, D.; Sadigh Eteghad, S.; Fazlollahi, A.; Nejadghaderi, S.A.; Shaafi, S. Association between miRNA-145 and miRNA-155 expression in peripheral blood mononuclear cells of patients with multiple sclerosis: a case-control study. *BMC Neurol* **2022**, *22*, 405, doi:10.1186/s12883-022-02909-6
405
10.1186/s12883-022-02909-6 [pii]
2909 [pii].
23. Keller, A.; Leidinger, P.; Lange, J.; Borries, A.; Schroers, H.; Scheffler, M.; Lenhof, H.P.; Ruprecht, K.; Meese, E. Multiple sclerosis: microRNA expression profiles accurately differentiate patients with relapsing-remitting disease from healthy controls. *PLoS One* **2009**, *4*, e7440, doi:10.1371/journal.pone.0007440
e7440
09-PONE-RA-12459R1 [pii].
24. Li, Y.C.; Chen, Y.; Liu, W.; Thadhani, R. MicroRNA-mediated mechanism of vitamin D regulation of innate immune response. *J Steroid Biochem Mol Biol* **2014**, *144 Pt A*, 81-86, doi:S0960-0760(13)00188-X [pii]
10.1016/j.jsbmb.2013.09.014.
25. Hanwell, H.E.; Vieth, R.; Cole, D.E.; Scillitani, A.; Modoni, S.; Frusciante, V.; Ritrovato, G.; Chiodini, I.; Minisola, S.; Carnevale, V. Sun exposure questionnaire predicts circulating 25-hydroxyvitamin D concentrations in Caucasian hospital workers in southern Italy. *J Steroid Biochem Mol Biol* **2010**, *121*, 334-337, doi:10.1016/j.jsbmb.2010.03.023

S0960-0760(10)00114-7 [pii].
26. Hedlund, L.; Brekke, H.K.; Brembeck, P.; Augustin, H. A Short Questionnaire for Assessment of Dietary Vitamin D Intake. *Eur J Nutr Food Saf* **2014**, *4*, 150-156.
27. Livak, K.J.; Schmittgen, T.D. Analysis of relative gene expression data using real-time quantitative PCR and the 2(-Delta Delta C(T)) Method. *Methods* **2001**, *25*, 402-408, doi:10.1006/meth.2001.1262
S1046-2023(01)91262-9 [pii].
28. Bates, D.; Mächler, M.; Bolker, B.; Walker, S. Fitting Linear Mixed-Effects Models Using lme4. *J of Stat Software* **2015**, *67*, 1-48, doi:https://doi.org/10.18637/jss.v067.i01.
29. Anderson, C.A.; Pettersson, F.H.; Clarke, G.M.; Cardon, L.R.; Morris, A.P.; Zondervan, K.T. Data quality control in genetic case-control association studies. *Nat Protoc* **2010**, *5*, 1564-1573, doi:10.1038/nprot.2010.116
nprot.2010.116 [pii].
30. Das, S.; Forer, L.; Schonherr, S.; Sidore, C.; Locke, A.E.; Kwong, A.; Vrieze, S.I.; Chew, E.Y.; Levy, S.; McGue, M.; et al. Next-generation genotype imputation service and methods. *Nat Genet* **2016**, *48*, 1284-1287, doi:10.1038/ng.3656.
31. Chang, C.C.; Chow, C.C.; Tellier, L.C.; Vattikuti, S.; Purcell, S.M.; Lee, J.J. Second-generation PLINK: rising to the challenge of larger and richer datasets. *Gigascience* **2015**, *4*, 7, doi:10.1186/s13742-015-0047-8
7
47 [pii].
32. Zhang, F.; Boerwinkle, E.; Xiong, M. Epistasis analysis for quantitative traits by functional regression model. *Genome Res* **2014**, *24*, 989-998, doi:10.1101/gr.161760.113
gr.161760.113 [pii].
33. Sticht, C.; De La Torre, C.; Parveen, A.; Gretz, N. miRWalk: An online resource for prediction of microRNA binding sites. *PLoS One* **2018**, *13*, e0206239, doi:10.1371/journal.pone.0206239
e0206239
PONE-D-18-17953 [pii].
34. Hsu, J.B.; Chiu, C.M.; Hsu, S.D.; Huang, W.Y.; Chien, C.H.; Lee, T.Y.; Huang, H.D. miRTar: an integrated system for identifying miRNA-target interactions in human. *BMC Bioinformatics* **2011**, *12*, 300, doi:10.1186/1471-2105-12-300
1471-2105-12-300 [pii].
35. Altschul, S.F.; Madden, T.L.; Schaffer, A.A.; Zhang, J.; Zhang, Z.; Miller, W.; Lipman, D.J. Gapped BLAST and PSI-BLAST: a new generation of protein database search programs. *Nucleic Acids Res* **1997**, *25*, 3389-3402, doi:gka562 [pii]
10.1093/nar/25.17.3389.
36. Gorenjak, M.; Repnik, K.; Jezernik, G.; Jurgec, S.; Skok, P.; Potocnik, U. Genetic prediction profile for adalimumab response in Slovenian Crohn's disease patients. *Z Gastroenterol* **2019**, *57*, 1218-1225, doi:10.1055/a-0981-6516.
37. Consortium, G. Erratum: Genetic effects on gene expression across human tissues. *Nature* **2018**, *553*, 530, doi:10.1038/nature25160
nature25160 [pii].
38. Tonacci, A.; Bagnato, G.; Pandolfo, G.; Billeci, L.; Sansone, F.; Conte, R.; Gangemi, S. MicroRNA Cross-Involvement in Autism Spectrum Disorders and Atopic Dermatitis: A Literature Review. *J Clin Med* **2019**, *8*, doi:10.3390/jcm8010088
88
jcm8010088 [pii]
jcm-08-00088 [pii].
39. Aljawadi, Z.A.; Al-Derzi, A.R.; Abdul-Majeed, B.A.; Almahdawi, A.M. MicroRNAs (20a, 146a, 155, and 145) expressions in a sample of Iraqi patients with multiple sclerosis. *J Fac Med Baghdad* **2016**, *58*, 371-377.
40. Hu, X.; Li, M.; Zhang, Y.; Sang, K.; Li, W.; Liu, B.; Wan, L.; Du, B.; Qian, J.; Meng, F.; et al. An innovative immunotherapeutic strategy for rheumatoid arthritis: Selectively suppressing angiogenesis and osteoclast differentiation by fully human antibody targeting thymocyte antigen-1. *Exp Cell Res* **2023**, *424*, 113490, doi:S0014-4827(23)00037-X [pii]
10.1016/j.yexcr.2023.113490.
41. Mithal, A.; Wahl, D.A.; Bonjour, J.P.; Burckhardt, P.; Dawson-Hughes, B.; Eisman, J.A.; El-Hajj Fuleihan, G.; Josse, R.G.; Lips, P.; Morales-Torres, J. Global vitamin D status and determinants of hypovitaminosis D. *Osteoporos Int* **2009**, *20*, 1807-1820, doi:10.1007/s00198-009-0954-6.
42. Zomot, E.; Achildiev Cohen, H.; Dagan, I.; Militsin, R.; Palty, R. Bidirectional regulation of calcium release-activated calcium (CRAC) channel by SARAF. *J Cell Biol* **2021**, *220*, doi:10.1083/jcb.202104007
e202104007
212731 [pii]
1. jcb.202104007 [pii].
2. 43. Taha, S.; Aljishi, M.; Alsharoqi, I.; Bakhiet, M. Differential upregulation of the hypothetical transmembrane protein 66 (TMEM66) in multiple sclerosis patients with potential inflammatory response. *Biomed Rep* **2015**, *3*, 98-104, doi:br-03-01-0098 [pii]

3. 10.3892/br.2014.390.
4. 44. Macian, F. NFAT proteins: key regulators of T-cell development and function. *Nat Rev Immunol* **2005**, *5*, 472-484, doi:nri1632 [pii]
5. 10.1038/nri1632.
6. 45. Bhattacharyya, S.; Deb, J.; Patra, A.K.; Thuy Pham, D.A.; Chen, W.; Vaeth, M.; Berberich-Siebelt, F.; Klein-Hessling, S.; Lamperti, E.D.; Reifenberg, K.; et al. NFATc1 affects mouse splenic B cell function by controlling the calcineurin--NFAT signaling network. *J Exp Med* **2011**, *208*, 823-839, doi:10.1084/jem.20100945
7. jem.20100945 [pii]
8. 20100945 [pii].
9. 46. Vaeth, M.; Muller, G.; Stauss, D.; Dietz, L.; Klein-Hessling, S.; Serfling, E.; Lipp, M.; Berberich, I.; Berberich-Siebelt, F. Follicular regulatory T cells control humoral autoimmunity via NFAT2-regulated CXCR5 expression. *J Exp Med* **2014**, *211*, 545-561, doi:10.1084/jem.20130604
10. jem.20130604 [pii]
11. 20130604 [pii].
12. 47. Vaeth, M.; Feske, S. NFAT control of immune function: New Frontiers for an Abiding Trooper. *F1000Res* **2018**, *7*, 260, doi:10.12688/f1000research.13426.1
13. 260.
14. 48. Chen, Y.; Liu, W.; Sun, T.; Huang, Y.; Wang, Y.; Deb, D.K.; Yoon, D.; Kong, J.; Thadhani, R.; Li, Y.C. 1,25-Dihydroxyvitamin D promotes negative feedback regulation of TLR signaling via targeting microRNA-155-SOCS1 in macrophages. *J Immunol* **2013**, *190*, 3687-3695, doi:10.4049/jimmunol.1203273
15. jimmunol.1203273 [pii].
16. 49. Saridas, F.; Tezcan Unlu, H.; Cecener, G.; Egeli, U.; Sabour Takanlou, M.; Sabour Takanlou, L.; Tunca, B.; Zarifoglu, M.; Turan, O.F.; Taskapilioglu, O. The expression and prognostic value of miR-146a and miR-155 in Turkish patients with multiple sclerosis. *Neurol Res* **2022**, *44*, 217-223, doi:10.1080/01616412.2021.1975221.
17.

Disclaimer/Publisher's Note: The statements, opinions and data contained in all publications are solely those of the individual author(s) and contributor(s) and not of MDPI and/or the editor(s). MDPI and/or the editor(s) disclaim responsibility for any injury to people or property resulting from any ideas, methods, instructions or products referred to in the content.
